# Handle the Autism Spectrum Condition during Coronavirus (COVID-19) *Stay at Home* Period: Ten Tips for Helping Parents and Caregivers of Young Children

**DOI:** 10.3390/brainsci10040207

**Published:** 2020-04-01

**Authors:** Antonio Narzisi

**Affiliations:** Department of Child Psychiatry and Psychopharmacology, IRCCS Stella Maris Foundation, 56018 Pisa, Italy; antonio.narzisi@fsm.unipi.it

## 1. Introduction

COVID-19 has become pandemic [1] and many government decrees have declared restrictive measures in order to prevent its wider spread. For parents and children, staying at home is one of these measures. In this situation the handling of young children with special needs such as autism spectrum condition (ASC) could be challenging for families and caregivers. Usually these children have interventions for several hours a week at home with special therapists or in dedicated hospitals and institutes. However at the moment, due to contagion containment measures, both the families and the ASC children are not physically supported by their therapists and they cannot attend the outside interventions. These measures, necessary for the health of all of us, need to be carefully handled to avoid an increase in parental stress and an exacerbation of children’s behavioral problems. ASC is a severe multifactorial disorder characterized by an umbrella of specific peculiarities in the areas of the social communication, restricted interests, and repetitive behaviours [2]. The incidence of ASC is worldwide and recent epidemiological data estimated it to be higher than 1/100 [3,4]. The main aim of this editorial is to give some advice, summarized in 10 tips, to help families to handle children with ASC during this period.

## 2. The 10 Tips for Helping Parents and Caregivers of Young Children

### 2.1. Explain to Your Child What COVID-19 Is

Children with ASC have a concrete cognitive style and some of them can have serious verbal issues and show difficulties in phenomenological perception [5]. It is important to explain what COVID-19 is and why we all have to stay at home. The explanation has to be simple and concrete. For this purpose it is possible to appeal to augmentative alternative communication (AAC). It is also possible to ask for help from therapists in preparing a brief pamphlet titled ‘What is COVID-19?’ using individualized AAC strategies. For verbal young children the explanation should be supported with concept mapping to make it easier for the child to understand.

### 2.2. Structure Daily Life Activities

It is widely reported that children with ASC have executive functioning deficits [6] and they could show issues in planning their daily life activities, especially when their routine is broken. For this reason it is important, especially now, to structure daily life activities. The home is the unique setting in which activities take place. It would be useful to subdivide the daily activities, assigning a different room for each one of them. This structure can be useful not only for children with ASC who are low and/or middle functioning but also for those who are high functioning. This can be an activity to share with the entire family as a type of game. Using a blackboard, each member of the family can have his space to write the planned activities.

### 2.3. Handle Semi-Structured Play Activities

Children with ASC enjoy playing, but they can find some types of play difficult because of sensory issues or because they prefer structured or semi-structured activities [7].

During the day it will be important to handle play activities. These can be individual and/or shared. Choose activities that your child prefers. For example, LEGO therapy [8,9] could be a good solution for children with ASC who are low or high functioning. LEGO-based therapy is an increasingly popular social skills programme for children and young people with social communication problems such as ASC. It can be a semi-structured play activity shared with parents or siblings in a home setting [10].

### 2.4. Use of Serious Games

Serious games can be useful to improve social cognition and to recognize facial emotions, emotional gestures, and emotional situations in children with ASC [11]. Serious games can be a fundamental resource for ASC children. Many serious games are free and can be downloaded as an App for tablet and/or PC from specialized sites. Serious games could be an educational alternative to video games or the internet tout-court.

### 2.5. Shared Video Game and/or Internet Sessions with Parents

Video games and the internet are extremely attractive for children with ASC but they could become an absorbent interest [12], especially in this period when children are called to stay at home. It is not possible to avoid children playing with the computer but at the moment, when parents are also at home, it could be useful establish a rule whereby children are expected to share the video games/internet (with parents, siblings, or other caregiver). This could avoid a potential risk of isolation of the child and an internet addiction.

### 2.6. Implement and Share Special Interests with Parents

Special interests can be a characteristic of the people with ASC. There is a growing amount of evidence recognizing the potential benefits that special interests can bring [13]. Special interests have to be supported from parents and/or caregivers. Trains, maps, animals, comic books, geography, electronics, and history can be just a few of potential special interests. In this period in which parents and children stay at home they could plan some activities sharing these special interests.

### 2.7. Online Therapy for High-Functioning Children

It is well recognized that psychiatric vulnerabilities and/or comorbidities are high in children with ASC. Among these comorbidities anxiety disorder is one of the most reported [14]. Psychiatric comorbidities could contribute to a developmental breakdown especially in adolescence age. The actual state of alert for COVID-19 could be an event that is difficult to mentalize for children with ASC. For this reason, if the children were engaged in psychotherapy before the COVID-19 alert, it is very important that they continue it. Since many therapists have stopped their face-to-face therapy, it is strongly advised to continue the psychotherapy in an online video or audio modality with the same weekly appointments. It could reduce the anxiety, check the mood, and offer to the children a private space in which to talk with a specialist. 

### 2.8. Weekly Online Consultations for Parents and Caregivers

Parents of children with autism experience more stress and are more susceptible than parents of children with other disabilities [15]. At the moment, parents are alone in the handling of their children with ASD. This can represent a further high risk for their stress levels, which are already severely tried. For this reason it can be very useful to have the opportunity for a weekly online consultation with the therapists of their children. It is valid for parents of both low- and high-functioning children. In the case of low functioning, parents could share a brief home video with the therapists about the behavior of the children during free play or structured sessions at home. In the case of children who are high functioning the consultation could be a dialogical exchange focused on the most appropriate ways to manage this difficult time of COVID-19 alert and to update parents about the degree of coping strategies of the children.

### 2.9. Maintain Contact with the School 

A growing body of research supports the suggestion that the relationships which children form with their teachers and classmates have an impact on learning [16]. It is very important to dedicate a time slot for the homework. This is a routine that has to be maintained. For the maintenance of social contacts with the school companions it is suggested to have at least a weekly contact with one of the class companions. The modality of this contact should depend from the child’s preferences. It could be an online video for those that prefer it. For children with ASC who do not prefer to use video for online contacts they could be encouraged to write a letter to one of their school companions or to call them via phone [17]. For both children and parents, it is strongly encouraged to maintain contact with a special teacher online or by phone. 

### 2.10. Leave Spare Time

Children with ASC have to be stimulated, as pointed in tips 1–9, but it is also possible be leave them a proper quota of spare time during the day (e.g., take a short walk near the house).

In this period children could have an increase in stereotypies. This does not need to be a particular concern. At the moment, when habits are changing, the stress levels can be elevated for children with ASC and the increase of stereotypies could be the behavioral result of perceived stress. They will certainly not regress.

## 3. Conclusions

These suggestions are obviously not exhaustive but they could represent a useful help for parents and/or caregiver of children with ASC to handle the severe situation caused COVID-19 and to optimize the person–environment fit.

COVID-19 is questioning the routine of our young children with ASC and they are called to respect rules and habits that are not always understandable for them (i.e., disinfect your hands, do not touch your eyes or nose, and cover your mouth. They are also not able to see people they would like to meet and must stay at home). These changing routines could cause them profound suffering. For this reason we all (parents, therapists, and researchers) must be united and quickly establish new and functional routines to allow our young children to be safe and peaceful. As ASC experts we have to find different ways to be close to our patients and their families. 

I wish to conclude this editorial by citing and sharing a sentence from Italian colleagues currently engaged in the emergency medical system in Milano: “The Italian public health authorities has just started to fight a battle that must be won” [18].
